# Posteroanterior Cervical Transcutaneous Spinal Cord Stimulation: Interactions with Cortical and Peripheral Nerve Stimulation

**DOI:** 10.3390/jcm10225304

**Published:** 2021-11-15

**Authors:** Jaclyn R. Wecht, William M. Savage, Grace O. Famodimu, Gregory A. Mendez, Jonah M. Levine, Matthew T. Maher, Joseph P. Weir, Jill M. Wecht, Jason B. Carmel, Yu-Kuang Wu, Noam Y. Harel

**Affiliations:** 1 James J. Peters VA Medical Center, Bronx, NY 10468, USA; jwecht@student.nymc.edu (J.R.W.); ws2384@columbia.edu (W.M.S.); grace.famodimu@va.gov (G.O.F.); gregory.mendez1@va.gov (G.A.M.); jonah.levine@va.gov (J.M.L.); matthew.maher@va.gov (M.T.M.); jill.wecht@va.gov (J.M.W.); yu-kuang.wu@va.gov (Y.-K.W.); 2 Department of Health, Sport & Exercise Sciences, University of Kansas, Lawrence, KS 66045, USA; joseph.weir@ku.edu; 3 Department of Rehabilitation and Human Performance, Icahn School of Medicine at Mount Sinai, New York, NY 10029, USA; 4 Department of Orthopedic Surgery, Columbia University, New York, NY 10032, USA; jbc28@columbia.edu; 5 Department of Neurology, Icahn School of Medicine at Mount Sinai, New York, NY 10029, USA

**Keywords:** spinal cord stimulation, cervical spinal cord injury, motor evoked potentials

## Abstract

Transcutaneous spinal cord stimulation (TSCS) has demonstrated potential to beneficially modulate spinal cord motor and autonomic circuitry. We are interested in pairing cervical TSCS with other forms of nervous system stimulation to enhance synaptic plasticity in circuits serving hand function. We use a novel configuration for cervical TSCS in which the anode is placed anteriorly over ~C4–C5 and the cathode posteriorly over ~T2–T4. We measured the effects of single pulses of TSCS paired with single pulses of motor cortex or median nerve stimulation timed to arrive at the cervical spinal cord at varying intervals. In 13 participants with and 15 participants without chronic cervical spinal cord injury, we observed that subthreshold TSCS facilitates hand muscle responses to motor cortex stimulation, with a tendency toward greater facilitation when TSCS is timed to arrive at cervical synapses simultaneously or up to 10 milliseconds after cortical stimulus arrival. Single pulses of subthreshold TSCS had no effect on the amplitudes of median H-reflex responses or F-wave responses. These findings support a model in which TSCS paired with appropriately timed cortical stimulation has the potential to facilitate convergent transmission between descending motor circuits, segmental afferents, and spinal motor neurons serving the hand. Studies with larger numbers of participants and repetitively paired cortical and spinal stimulation are needed.

## 1. Introduction

Both invasive and non-invasive forms of repetitive electrical spinal cord stimulation have shown great promise in amplifying supraspinal influence over the sublesional cord after spinal cord injury (SCI) [1,2,3,4,5,6,7,8,9]. Notably, a non-invasive approach to spinal cord stimulation carries significantly lower risk with greater potential for widespread implementation, especially at the cervical level.

Transcutaneous spinal cord stimulation (TSCS) paradigms generally involve cathodal stimulation over the cord, with anodes usually placed over the iliac crests or abdomen [5,6,7,9]. We and others have demonstrated that single-pulse cervical TSCS can be safely performed using a posteroanterior configuration with the cathode placed over the upper thoracic spinous processes and the anode placed over the anterior surface of the neck [10,11]. This posteroanterior TSCS configuration easily elicits muscle responses across multiple cervical myotomes through a mix of sensory afferent and motor efferent circuit activation. At low stimulus intensities, posteroanterior TSCS appears to activate predominantly sensory afferent circuits, whereas at higher stimulus intensities, motor efferents are directly activated [11].

Sensory afferent fiber activation likely mediates the beneficial effects of both epidural and transcutaneous spinal cord stimulation [9,12,13,14,15]. We are interested in exploring the potential for pairing TSCS-mediated afferent activation with appropriately timed descending motor signals to enhance synaptic potentiation through heterosynaptic summation. In this study, we measured upper extremity muscle responses to single-pulse transcranial magnetic stimulation (TMS) conditioned with subthreshold cervical TSCS at varying interstimulus intervals in individuals with chronic cervical SCI and able-bodied volunteers. To provide further insight into posteroanterior TSCS effects on motor neuron excitability and synaptic transmission within the cervical cord, we also measured interactions between TSCS and peripheral nerve stimulation at either supramaximal (F-wave responses) or submaximal (H-reflex responses) stimulus intensity. We hypothesized that low-intensity posteroanterior cervical TSCS would increase upper extremity muscle responses to motor cortical stimulation in a timing-dependent manner.

## 2. Materials and Methods

### 2.1. Design

This prospective human research study was approved by the Institutional Review Board of the James J. Peters VA Medical Center, Bronx, NY (MIRB# 01743). All applicable institutional and governmental regulations concerning the ethical participation of human volunteers were followed during the course of this research. This manuscript reports data from TSCS-conditioning experiments registered at clinicaltrials.gov (NCT03414424).

### 2.2. Participants

Individuals between ages 21 and 75 without neurological injury (able-bodied or AB) and those with chronic cervical SCI were eligible for participation. For participants with SCI, inclusion criteria included duration of injury greater than 12 months, level of injury between C2 and C8, and incomplete paresis of intrinsic muscles in either hand. All participants required detectable F-wave responses of left or right abductor pollicis brevis (APB) muscle to median nerve stimulation or first dorsal interosseous (FDI) muscle to ulnar nerve stimulation, and detectable motor evoked potentials (greater than 50 μV) in left or right APB or FDI muscle to TMS. Exclusion criteria included ventilator dependence, open tracheostomy site or other open lesions over the neck, shoulders, or arms, multiple sclerosis, stroke, amyotrophic lateral sclerosis, or other serious neurological disorder, hemorrhagic brain injury, seizures, medications that increase seizure risk, self-reported recurrent spontaneous bouts of symptomatic autonomic dysreflexia, significant coronary artery or cardiac conduction disease, bipolar disorder, active psychosis, pregnancy, or implanted electrical or ferromagnetic devices [16]. Participant ID numbers were assigned in order of study enrollment.

### 2.3. General Protocol

Sessions were performed at a consistent time of day in each participant, with attempts to maintain consistent timing of caffeine intake if applicable. Stimulation was delivered with participants in an upright seated position in an adjustable TMS chair (Magventure) or in an individual’s personal wheelchair if preferred. Arms were flexed at roughly 90 degrees, with the hands pronated and relaxed on a pillow cushion placed in the participant’s lap. For AB participants, stimulation was targeted toward the dominant arm. For those with SCI, stimulation was targeted toward the arm with lower motor thresholds and more consistent electrophysiological responses to central and peripheral stimulation. Blood pressure, heart rate, pulse oximetry, and symptoms, such as a headache, chest tightness, shortness of breath, and palpitations, were monitored and recorded every three minutes during TSCS, and no less than every 15 min during other portions of the protocol. Subjective symptoms related to TMS such as headache, confusion, hearing loss, etc., were assessed according to questions suggested by the International Federation of Clinical Neurophysiology [17]. To further evaluate potential autonomic effects of TSCS, one session incorporated continuous beat-to-beat hemodynamic monitoring, described below.

### 2.4. Electromyography (EMG)

EMG was recorded using surface sensors with ×300 preamplification, 15–2000 Hz bandwidth, and internal grounding (Motion Lab Systems Z03-002). EMG was collected at a sample rate of 5000 Hz via digital acquisition board and customized LabVIEW software (National Instruments USB-6363). This manuscript reports data for the target arm APB and flexor carpi radialis (FCR). Note that two participants (#18 and #27) had unreliable APB responses, so the target arm first dorsal interosseous (FDI) was analyzed.

### 2.5. Transcutaneous Spinal Cord Stimulation (TSCS)

Stimulation was delivered using 5 × 10 cm electrodes (Natus 019-422200). The cathode electrode was placed longitudinally over the posterior midline with the cephalad edge ~4 cm caudal to the C7 spinous process, corresponding to the T2–T4 vertebral levels posteriorly. The anode electrode was placed horizontally over the anterior midline with the caudal edge ~2–3 cm superior to the sternal notch, corresponding to the C4–C5 levels anteriorly [11]. Two 5 × 10 cm electrodes over the distal clavicles were connected to a common ground.

Stimulation (2 ms biphasic pulses) was delivered using constant-current stimulators (Digitimer DS7A or DS8R). Resting motor threshold (RMT) was determined as the intensity (in mA) required to elicit a potential in the APB muscle of at least 50 µV in 5 out of 10 repetitions. All subsequent TSCS testing was performed at specified intensities normalized to each individual’s APB RMT.

### 2.6. Transcranial Magnetic Stimulation (TMS)

A MagPro X100 system (Magventure) with 80 mm winged coil (D-B80) was used. The magnet was oriented at a 45-degree angle from the sagittal plane, centered over the hand motor cortex hotspot for maximal APB response. Coil and hotspot positioning were tracked using an optical-based neural navigation system (Brainsight 2.4, Rogue Research, Montreal, QC, Canada). RMT was determined as the percent of maximal stimulator output required to elicit an MEP of at least 50 μV in the resting APB muscle in 5 out of 10 repetitions. All subsequent TMS testing was completed at specified percentages of TMS intensity normalized to each individual’s APB RMT.

### 2.7. Peripheral Nerve Stimulation (PNS)

Stimulation was delivered using constant-current stimulators (Digitimer DS7A or DS8R) via dual surface electrodes (Natus 019-429400) placed over the median nerve at the wrist (F-waves), or the median nerve at the elbow (H-reflex). For F-waves, monophasic 0.2 ms duration pulses were delivered at supramaximal intensity 25 times at 0.5 Hz to record both direct (M-wave) and late (F-wave) responses at the APB. The minimal F-wave latency was used to calculate the peripheral motor conduction time (PMCT) as (Latency_M_ + Latency_F_ − 1) ÷ 2 [18]. Central motor conduction time (CMCT) was calculated as MEP latency (at 120% of TMS RMT) minus PMCT. For H-reflexes, monophasic 1.0 ms duration pulses were delivered across a range of submaximal intensities at 0.2 Hz to determine the maximal and 50%-maximal H-reflex amplitudes at the FCR muscle. The H-reflex conduction time (HRCT) was calculated as (Latency_H_ − Latency_M_ − 1) ÷ 2.

F-wave persistence was calculated based on the percentage of total positive F-waves, defined as an F-wave with an amplitude above 20 μV. F-wave peak-to-peak amplitudes were normalized to the maximal compound motor action potential (CMAP) from that session [19].

### 2.8. Hemodynamic Data Collection

Seven AB volunteers and five participants with SCI underwent an extra experiment with continuous hemodynamic data collection. Prior to initiation of study procedures, participants were asked to empty their bladder and to loosen any tight-fitting clothing or belt. Although the participant rested quietly in the seated position, instrumentation was applied, which included: (1) a three-lead ECG and respiration monitor with electrodes placed at the right and left mid-axillary lines in the 5th intercostal space and at the right anterior axillary line (Model RESP 1 with EKG: UFI, Morro Bay, CA, USA), and (2) a finger BP monitor was placed on the index finger or middle finger of the non-dominant hand (AB) or the non-targeted hand (SCI) (Finapres Medical Systems, Amsterdam, Netherlands) for simultaneous assessment of continuous beat-to-beat BP and HR. After instrumentation, a 5-min baseline assessment of HR and BP was recorded in the seated position prior to stimulation. Beat-to-beat HR and BP were then recorded for 1 min prior to TSCS, during TSCS, and 1 min post TSCS. TSCS was delivered at three intensities relative to RMT in random order: one subthreshold (70%) and two suprathreshold (125% and 175%), at 0.1–0.2 Hz for 6–10 repetitions. There was a 1–2 min rest period between delivery of each TSCS intensity. Beat-to-beat BP and HR signals were sampled at 500 Hz using customized data acquisition programs written with LabVIEW software (version 2014 SP1, National Instruments, Austin, TX, USA). The raw BP and HR data files were stored for offline data analysis conducted using customized software programs written with LabVIEW graphical software.

### 2.9. TSCS-TMS Interactions

To test whether subthreshold TSCS could facilitate response to suprathreshold TMS (120% of motor threshold), single pulses of TMS were delivered either alone (control) or conditioned with single pulses of TSCS delivered across a range of intensities and interstimulus intervals. TSCS was delivered at 50%, 70%, or 90% RMT, timed to arrive at cervical synapses at intervals ranging from 25 ms prior to TMS arrival to 10 ms after TMS arrival (Table 1). Cervical cord arrival timing was calculated utilizing participant-specific CMCT and PMCT, with subthreshold TSCS conduction time set at 1.5–2 ms for all participants [11,20,21]. Unpaired TSCS pulses were delivered as further controls. Paired or unpaired pulses were delivered at 0.1 Hz in pseudorandom order. Each condition was repeated 8 times per session except for unconditioned TMS, which was repeated 16 times in session 1 and 10 times in session 2. To reduce participant burden (nearly 1000 paired pulse paradigms delivered across experiments), we prioritized more repetitions of unconditioned TMS, as this was the control against which all paired TSCS-TMS paradigms were compared. A subset of the paired TSCS-TMS paradigms was repeated on two separate days to accumulate more repetitions.

### 2.10. TSCS-PNS Interactions: F-Waves

PNS was delivered over the median nerve at the wrist of the target hand at supramaximal intensity, recording over the APB muscle (except over the ulnar nerve at the wrist, recording over the FDI muscle in two participants (#18 and #27)). PNS was delivered either alone (control) or conditioned with single pulses of TSCS delivered across a range of intensities and interstimulus intervals. TSCS was delivered at 50%, 70%, or 175% RMT. Subthreshold TSCS pulses were timed to arrive at cervical synapses at intervals ranging from 200 ms to 2 ms prior to PNS arrival, or simultaneously with PNS arrival at cervical motor neurons (Table 2). Suprathreshold TSCS pulses were delivered to either arrive at cervical motor neurons 10 ms prior to or simultaneously with PNS arrival to test for collisional interference or facilitation of F-wave responses, respectively [21]. Unpaired TSCS pulses were delivered as further controls. Paired or unpaired pulses were delivered at 0.1 Hz in pseudorandom order. This experiment was repeated on two separate days with partially overlapping conditions to confirm reliability. Each condition was repeated 25 times per session except for unconditioned TSCS, which was repeated 8 times in session 1 and 7 times in session 2.

### 2.11. TSCS-PNS Interactions: H-Reflexes

PNS was delivered over the median nerve at the elbow of the target hand, recording at the FCR muscle. PNS intensity was calibrated to result in H-reflex amplitude ~50% of H_max_. PNS was delivered either alone (control) or conditioned with single pulses of TSCS stimuli (50% RMT intensity) timed to arrive at cervical synapses at intervals ranging from 100 ms to 2 ms prior to PNS arrival, or simultaneously with PNS arrival (Table 2). Unpaired TSCS pulses were delivered as further controls. Paired or unpaired pulses were delivered at 0.1 Hz in pseudorandom order. Each condition was repeated 10 times during one session.

### 2.12. Data Analysis

Pulse responses were discarded if spontaneous muscular activity was observed within the 50 ms prior to stimulation, if the participant was noted to move during a stimulation, if background electrical noise was above 50 μV, or if the TMS coil was noted to be off-target during experimentation.

Peak-to-peak amplitudes were quantified for all responses to TSCS, TMS, and peripheral nerve stimulation. For conditioning paradigms, each participant’s amplitudes of TSCS-conditioned responses (or unpaired TSCS responses) were normalized to that participant’s average amplitudes for unconditioned TMS, F-wave, or H-reflex responses. Furthermore, due to the definition of RMT as the intensity at which half of stimuli evoke a response of >50 µV, some TSCS stimuli at 70% and 90% of threshold evoked detectable responses. If at a given TSCS intensity, the average response amplitude across multiple pulses per session was greater than 30% of the response to TMS, then all TSCS-conditioned responses to TMS at that intensity were discarded for that session.

### 2.13. Statistical Analysis

Data are reported as the mean ± standard error of the mean. Due to non-normal data distributions, non-parametric Mann-Whitney U Tests were used to compare differences between AB and SCI groups for response amplitudes to TMS alone, TSCS alone, and peripheral nerve stimulation (F or H) alone.

For the TSCS conditioning paradigms, linear mixed modeling was performed using a maximum likelihood estimation approach. Fixed effects were: group (AB vs. SCI), TSCS intensity (50%, 70%, 90%, and/or 175%), and synaptic interval (7 levels ranging from −200 ms to +10 ms depending on paradigm). Participants and sessions were random effects. Main and interaction effects were subjected to analysis of variance (ANOVA) using Satterthwaite’s method. Significance was set at an alpha level of 0.05.

Excel (Microsoft, Redmond, Washington, DC, USA), SPSS Version 28 (IBM, Armonk, New York, NY, USA), and R (https://www.R-project.org/, accessed on 4 October 2021) were used for all analyses.

Individual-level data are included as Supplementary Tables S2–S8.

## 3. Results

### 3.1. Participants

In total, 30 participants (15 AB, 15 SCI; 23 males, 7 females) enrolled, and 28 (15 AB, 13 SCI) passed screening (Table 2). The groups did not differ significantly for age, which ranged from 22 to 71 years old. Of the 13 SCI participants, 12 had traumatic SCI, one had idiopathic transverse myelitis. Data for TMS were excluded from two participants (23 and 27) who were found after screening to either have unacceptable electrical background EMG activity or unreliable responses in resting muscle during TMS. Thus, analysis of TMS results included 15 AB and 11 SCI participants, whereas analysis of TSCS and PNS results included 15 AB and 13 SCI participants.

### 3.2. TMS Responses

**Figure 1:**
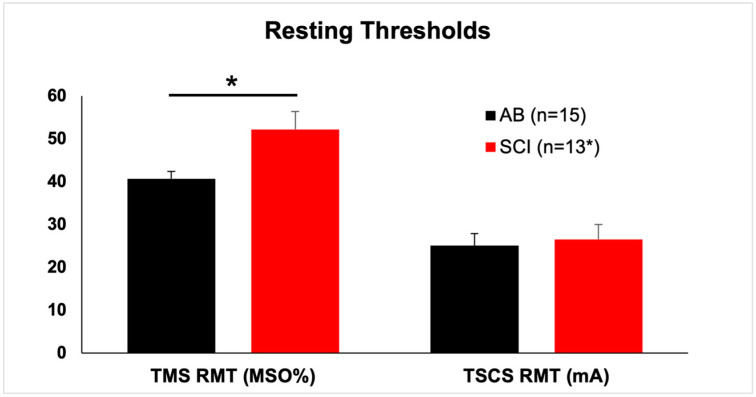
Resting thresholds are higher in SCI participants for TMS but not TSCS. Resting motor threshold (RMT) for the abductor pollicis brevis (in two SCI participants, the first dorsal interosseous). Note that two SCI participants had unobtainable transcranial magnetic stimulation (TMS) responses, whereas all participants responded to transcutaneous spinal cord stimulation (TSCS). Hence, the asterisk next to “13” in the legend. MSO%, percent of maximal stimulator output. mA, milliamperes. Mean and SEM shown. *, *p* = 0.024.

SCI participants showed significantly higher mean ± SEM RMT at the APB muscle (52.2% ± 4.2% maximum stimulator output) than AB participants (40.7% ± 1.7% maximum stimulator output) (*p* = 0.024, Mann-Whitney U test) (Figure 1; Table 3).

SCI participants showed significantly lower mean ± SEM amplitudes at 120% of RMT (0.183 mV ± 0.057 mV) than AB participants (0.549 mV ± 0.051 mV) (*p* < 0.001, Mann-Whitney U test).

### 3.3. TSCS Responses

As observed in our prior work, there was no difference in TSCS response thresholds between AB and SCI participants. Mean ± SEM TSCS RMT at the APB muscle was 25.1 mA ± 2.8 mA for AB participants and 26.5 ± 3.5 mA for SCI participants (non-significant, Mann-Whitney U test) (Figure 1; Table 3).

### 3.4. PNS (F-Wave) Responses

SCI participants showed larger amplitude, more persistent F-waves than AB participants. Relative to M_max_, the amplitude of unconditioned F-waves was 0.019 ± 0.002 for AB participants and 0.093 ± 0.027 for SCI participants (*p* = 0.014, Mann-Whitney U test). Correspondingly, mean F-wave persistence tended to be lower for AB participants (68.1% ± 4.9%) than for SCI participants (77.0% ± 9.3%) (*p* = 0.069, Mann-Whitney U test) (Table 3).

### 3.5. PNS (H-Reflex) Responses

Only 7 AB and 6 SCI participants had reliable FCR H-reflex responses. Relative to M_max_, maximal H-reflex amplitude was similar in AB (0.293 ± 0.063) and SCI participants (0.229 ± 0.082) (non-significant, Mann-Whitney U test) (Table 3).

### 3.6. TSCS-TMS Interactions

**Figure 2:**
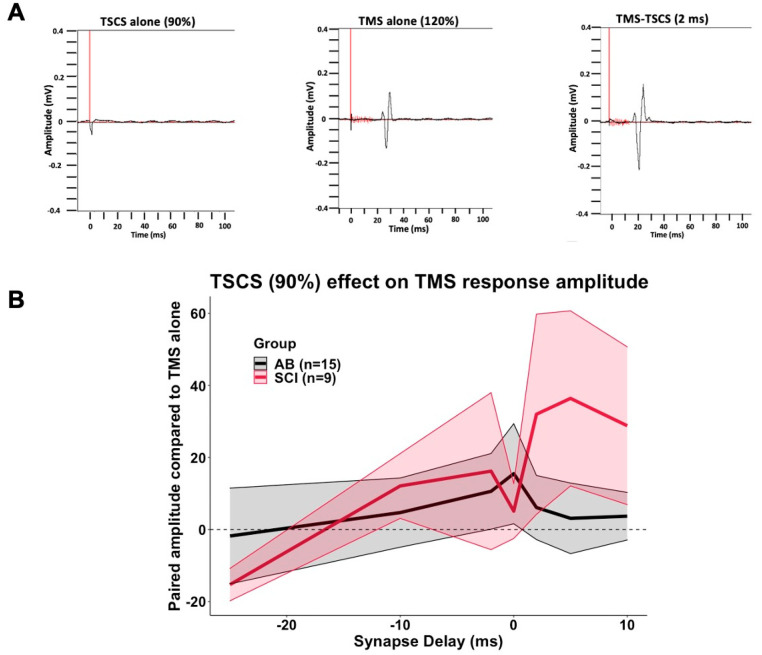
Subthreshold TSCS acutely facilitates TMS-evoked potentials. Suprathreshold (120%) TMS and subthreshold TSCS were given alone or in combination as depicted in Table 1. Response amplitudes were compared to the response to TMS alone (0). Synapse delay represents the time of TSCS pulse arrival at cervical motor neurons relative to test pulse arrival. Negative numbers indicate TSCS pulse arrival before test pulse arrival. (**A**). Representative waves from participant #29. (**B**). Effect of conditioning TSCS at 90% RMT. Black line (grey shading) indicates mean (SEM) for AB participants. Red line (pink shading) indicates mean (SEM) for SCI participants.

TMS-evoked APB muscle amplitudes were facilitated by subthreshold TSCS, with a significant main effect of TSCS intensity (F = 4.401, *p* = 0.013) and synapse delay (F = 2.520, *p* = 0.020) but no main effect of group (F = 0.268, *p* = 0.607). There was significant interaction between group and TSCS intensity (F = 4.205, *p* = 0.015) but not between group and synapse delay (F = 0.583, *p* = 0.744) or TSCS intensity and synapse delay (F = 1.174, *p* = 0.297). Though not statistically significant on pairwise comparisons, TSCS-mediated facilitation tended to be stronger when TSCS pulses arrived at cervical synapses simultaneously or up to 10 ms after TMS pulse arrival (Figure 2, Table 4). Increasing TSCS intensity tended to facilitate TMS responses more in SCI than in AB participants.

### 3.7. TSCS-PNS Interactions: F-Waves

Subthreshold TSCS did not substantially affect F-wave response amplitudes in either AB or SCI participants (Table 5). There was no main effect of TSCS intensity (F = 0.377, *p* = 0.540) or group (F = 3.612, *p* = 0.062). There was a main effect of synapse delay (F = 2.148, *p* = 0.048). However, there were no interaction effects, and no significant effect of any individual synapse delay on pairwise comparisons. Subthreshold TSCS tended to have more effect on F-wave persistence than amplitude. There was a main effect of synapse delay (F = 2.543, *p* = 0.021) and an interaction effect between group and synapse delay (F = 2.684, *p* = 0.015) but no main effect of group (F = 0.898, *p* = 0.347) or intensity (F = 0.186, *p* = 0.666). There was no significant effect of any individual synapse delay on pairwise comparisons.

**Figure 3:**
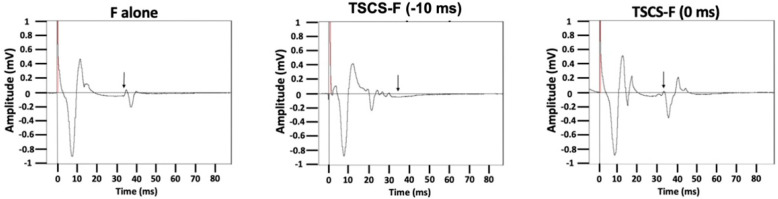
Suprathreshold TSCS collides with or facilitates F-wave responses depending on timing. Supramaximal median nerve stimulation was delivered to generate F-wave responses. Conditioning TSCS pulses at 175% of RMT were timed to arrive at cervical motor neurons either 10 ms prior to or simultaneously with retrograde median nerve pulse arrival.

Suprathreshold (175% RMT) TSCS, previously shown to directly activate motor efferents [11], facilitated an F-wave response when TSCS arrived at cervical motor neurons simultaneously to retrograde F-wave arrival. Suprathreshold TSCS interfered with F-wave transmission when TSCS was timed to arrive at cervical motor neurons 10 ms prior to retrograde F-wave arrival (Figure 3). The difference in amplitude and in persistence between simultaneous and early (collisional) TSCS arrival was significant across both AB and SCI participants: main effect of synapse delay (F = 9.879, *p* = 0.003 for amplitude; F = 19.804, *p* < 0.0005 for persistence); no main effect of group or group × synapse delay interaction.

### 3.8. TSCS-PNS Interactions: H-Reflexes

Subthreshold TSCS did not significantly affect H-reflex responses at any synaptic interval in either AB or SCI participants (Supplementary Table S1).

### 3.9. Safety and Hemodynamic Responses

No serious adverse events occurred during this study. The most common mild adverse events were transient headache and neck soreness (5 incidents each). These symptoms were more often related to TMS than to TSCS.

Seven AB and five SCI participants underwent hemodynamic monitoring while receiving TSCS at 70%, 125%, or 175% of RMT. Results of repeated measures ANOVA indicated no significant main or interaction effects for condition (intensity) or group (AB, SCI) on heart rate or systolic BP (Supplementary Figure S1). The largest change in BP (a decrease of, roughly, 18 mm Hg systolic) was noted in AB #6, a 22 year-old man with no neurological history. This participant moved his hands excessively during the experiment, which interfered with the beat-to-beat recording on the 3rd digit of his non-target hand (see Methods) and caused an artifactual drop in BP readings.

## 4. Discussion

Repetitive stimulation of the brain, spinal cord, and/or peripheral nerves has long been known to affect ensuing muscle activity. The optimal combination of timing, intensity, frequency, waveforms, and participant characteristics have yet to be determined despite numerous studies. Far less study has been directed toward paired stimulation at two sites of the nervous system to magnify the muscular response to stimulation. Paired associative stimulation (PAS) times brain and peripheral nerve stimuli to converge in sensorimotor cortex [22,23,24]. Rather than cortical convergence, studies have also demonstrated that spinal convergence of segmental stimuli with descending cortical stimuli may have summative effects. In the lumbar spine, suprathreshold TSCS, presumably activating dorsal sensory afferent fibers, facilitated responses in the soleus and hamstring muscles but not tibialis anterior when spinal impulses temporally converged at spinal motor neurons with cortical impulses [25]. Convergent arrival at spinal motor neurons of suprathreshold afferent peripheral stimuli and descending stimuli from TMS leads to summation of responses at several hand [26] and leg muscles [27]. A related paradigm (spike timing-dependent plasticity) in which peripheral stimulation is delivered retrogradely through motor axons timed to converge in the spinal cord with descending cortical stimulation also facilitates hand [20,28,29] and leg [30,31] responses. Invasive experiments in rhesus monkeys further support the concept of facilitating spinal motor neuron responses through heterosynaptic summation partly through dorsal afferent spinal input [32,33].

To increase the applicability of spinal stimulation toward physical rehabilitation protocols, spinal stimulation needs to be delivered at subthreshold intensities, (1) so as not to interfere with ongoing movements, and (2) because noxious suprathreshold stimuli disrupt motor learning [34]. Multiple studies in rodents have shown that subthreshold spinal stimulation facilitates upper limb responses to motor cortex stimulation [15,35,36,37], though not with uniform results [38]. Furthermore, a recent study of spinal stimulation combined with volitional handgrip exercise in healthy humans showed that subthreshold stimulation achieved greater facilitation of spinal and corticospinal responses than suprathreshold stimulation [39]. To our knowledge, the current study is the first experiment in humans with and without SCI to measure the effect of paired suprathreshold cortical with subthreshold cervical spinal cord stimulation across a range of interstimulus intervals. This study intended to further our understanding of how cervical TSCS delivered in a novel posteroanterior configuration [10,11] interacts with other forms of peripheral and central nervous system stimulation, and to explore the potential for using subthreshold cervical TSCS to facilitate motor responses to brain stimulation.

Based on the studies cited above, we hypothesized that subthreshold TSCS can amplify hand muscle responses to motor cortex stimulation through heterosynaptic summation [24,25]. We expected single pulses of subthreshold TSCS to increase the amplitude of hand muscle responses to single pulses of suprathreshold motor cortex TMS when the pulses were timed to temporally converge at cervical motor synapses. Furthermore, we expected that single pulses of subthreshold TSCS would not affect lower motor neuron responses to F-wave stimulation (non-synaptic), and that single pulses of subthreshold TSCS would reduce the FCR response to H-reflex stimulation (synaptic). These findings would support a model in which TSCS facilitates convergent transmission between descending motor circuits, segmental afferents, and spinal motor neurons rather than directly affecting intrinsic spinal motor neuron excitability [25,40].

Our findings in this study did not fully confirm our hypotheses. Though TMS responses tended to be higher when afferent TSCS arrived at cervical cord synapses simultaneously to up to 10 milliseconds after TMS, these effects were somewhat variable and not statistically significant. For intervals up to ~2 ms between TMS and subsequent electrical signal arrival at the cervical cord, there is established evidence of associative facilitation [15,20,28,37]. For 5–10 ms intervals, we speculate that the arriving TSCS pulse interacts with the TMS volley of indirect waves in a way that facilitates the resulting MEP [40]. One major technical factor that affected the consistency of our results was the way in which RMT was defined. Analogous to the method for determining TMS RMT, we defined TSCS RMT as the intensity required to produce a response >50 μV in 5 out of 10 trials. We often see responses of 20–40 μV to stimuli at 90% and even 70% of RMT, the main intensities of interest used in this study. Thus, even though we discarded data when TSCS responses were greater than 30% of the TMS response, we cannot exclude a non-specific partially suprathreshold facilitation across a wider range of interstimulus intervals than intended [25].

In an unpaired stimulation, we, unsurprisingly, observed higher TMS thresholds and lower TMS-evoked muscle response amplitudes in SCI participants and confirmed our prior findings that TSCS thresholds are similar in AB and SCI participants [11]. We also observed higher F-wave amplitude and persistence in SCI participants than in AB participants. This suggests an increased state of spinal motor neuron excitability after SCI, as has been noted by others [41], especially in the context of lesions in the rostral cervical cord [42]—of the 13 participants with SCI in our study, 6 had SCI at C3, and 2 had SCI at C4. Conversely, we observed a non-significantly higher unpaired H-reflex amplitude in AB participants than in SCI participants, which was surprising given that H-reflexes are usually increased below the level of SCI.

Results of pairing subthreshold TSCS with F-wave responses were mixed. Overall, synapse delay between TSCS and F-wave arrival at the cervical cord appeared to reduce F-wave amplitude and persistence in SCI but not AB participants, though this did not reach significance on pairwise comparisons. We speculate this may have resulted from the higher baseline F-wave amplitude and persistence in SCI participants. The lack of a consistent effect of subthreshold TSCS on F-wave responses corresponds to findings that vibratory stimuli activating Ia afferents do not affect F-waves [43], and is consistent with a model that at low stimulus intensity, TSCS activates dorsal afferent fibers, modulating spinal motor neurons through indirect transsynaptic pathways [10,11,25,44].

Our results with suprathreshold TSCS shed more insight into routes of posteroanterior TSCS transmission. Our previous data had shown that as posteroanterior cervical TSCS intensity increased, post-activation depression of ensuing pulses decreased, suggesting more efferent motor fiber activation [11]. The finding in the current study that TSCS delivery at 175% of RMT 10 ms prior to peripheral F-wave stimulation reduced F-wave amplitude and persistence indicates collisional interference between the anterograde TSCS pulse and retrograde F-wave along motor axons [21]. Likewise, when the interstimulus interval was adjusted for convergent arrival of suprathreshold TSCS and retrograde F-wave at spinal motor neurons, the F-wave amplitude and persistence increased. Both of these findings further support the model that high-intensity posteroanterior cervical TSCS directly activates motor nerve roots.

H-reflex transmission is carried by Ia afferents that synapse with segmental spinal motor neurons. These synapses are susceptible to homosynaptic and post-activation depression, i.e., when H-reflex stimuli are delivered in intervals of roughly five seconds or less, responses are blunted after the first response [9,10,45]. Multiple groups have shown that at most intensities, especially subthreshold intensity, TSCS also activates large-diameter dorsal afferent fibers, and are alternatively termed posterior root reflexes [44]. In this study, TSCS pulses were delivered 200 ms or less prior to H-reflex pulses, well within the window of post-activation depression. Hence, since TSCS pulses presumably emulate H-reflex transmission, we thus expected conditioning subthreshold TSCS to decrease H-reflex amplitudes across all interstimulus intervals in both AB and SCI populations. A reduction in H-reflex amplitude would be clinically relevant in the SCI population, as more than 60% of people with SCI experience spasticity [46,47]. In fact, repetitive TSCS of the lumbar spine has been shown to reduce spasticity after SCI [48,49]. However, we did not see significant changes in H-reflex amplitudes when conditioned with TSCS in the current study. Only 7 AB and 6 SCI participants demonstrated easily distinguishable FCR H-reflexes in this study, a number prone to either Type I or Type II error and too small to make definitive conclusions. To reduce participant burden in terms of overall number of pulses delivered, the TSCS-H-reflex conditioning experiments in our study only applied TSCS at 50% of RMT, possibly at too low an intensity to achieve post-activation depression.

### 4.1. Limitations

Multiple limitations affected this study. The small sample size limited our ability to determine reliable associations between injury characteristics and results of different stimulation paradigms. Conditioning experiments involving TSCS paired with peripheral nerve stimulation predominantly applied TSCS at only 50% of RMT, perhaps too low of an intensity to mediate significant effects. Conversely, as discussed earlier, the traditional definition of motor threshold we used (a response of >50 μV in 5 out of 10 trials) resulted in ‘subthreshold’ TSCS pulses leading to action potentials in several participants, complicating our ability to focus on subthreshold TSCS conditioning. To address this, we discarded data from sessions in which a participant’s response amplitude to unconditioned TSCS was greater than 30% of unconditioned TMS amplitude. To reduce participant burden while undergoing multiple different conditioning paradigms, many of the TSCS–TMS paradigms were only measured 8 times per participant, further increasing variability [50]. Likewise, we did not test interstimulus intervals in which TSCS arrived at cervical spinal synapses greater than 10 ms after TMS arrival-in retrospect, this prevented us from mapping a full curve of increased and decreased facilitation across interstimulus intervals in the TSCS-conditioned TMS experiments.

### 4.2. Further Information and Experiments Needed

Though we focused on the APB in this study and normalized all stimulus intensities to APB motor thresholds, we recorded other muscles in both upper extremities. This raw data have yet to be fully analyzed and will be disseminated separately. The effects of TSCS and TSCS-conditioned TMS on autonomic parameters, such as blood pressure and cerebral blood flow, need to be carefully measured. Future experiments incorporating subthreshold TSCS should probably use a different, less stringent definition for motor threshold than traditionally used in TMS experiments. For example, an amplitude cutoff of 20 μV rather than 50 μV, or a cutoff of 2 rather than 5 positive responses out of 10 threshold trials.

## 5. Conclusions

We measured the effects of single pulses of posteroanterior cervical TSCS paired with single pulses of motor cortex or median nerve stimulation timed to arrive at the cervical spinal cord at varying intervals. In 13 participants with and 15 participants without chronic cervical spinal cord injury, we observed that subthreshold TSCS facilitates hand muscle responses to motor cortex stimulation, with a tendency toward greater facilitation when TSCS is timed to arrive at cervical synapses simultaneously or up to 10 milliseconds after cortical stimulus arrival. When paired with median nerve stimulation, single pulses of subthreshold TSCS had no effect on the amplitude of H-reflex responses or F-wave responses. Though variability was high, the overall findings suggest that TSCS paired with appropriately timed cortical stimulation has the potential to facilitate convergent transmission between descending motor, segmental afferent, and spinal motor neurons serving the hand. Since TMS travels via similar descending motor pathways that mediate volitional movement, studying TSCS-conditioned TMS responses can produce findings with potential for direct clinical translation. To improve reliability, further studies with larger numbers of participants and repetitively paired cortical and cervical spinal stimulation are needed.

## Figures and Tables

**Table 1:** Scheme for conditioning experiments. Conditioning TSCS pulses were delivered at the indicated intensities (in % of TSCS resting motor threshold) at various synaptic delays relative to test pulses (TMS, F-wave, or H-reflex). Synaptic delay represents the time of TSCS pulse arrival at cervical motor neurons relative to test pulse arrival. Negative numbers indicate TSCS pulse arrival before test pulse arrival.

Synaptic Delay (ms)	TSCS Intensity (% RMT)		
	TMS	F	H
−200		50	
−100			50
−50		50	50
−25	50, 70, 90	50	50
−10	50, 70, 90	50, 70, 175	50
−5		50, 70	50
−2	50, 70, 90	50, 70	50
0	50, 70, 90	50, 175	50
2	50, 70, 90		
5	50, 70, 90		
10	50, 70, 90		

TSCS: Transcutaneous spinal cord stimulation; RMT: Resting motor threshold; TMS, transcranial magnetic stimulation; F, F-wave; H, H-reflex.

**Table 2:** Participant demographics. NT—non-traumatic; DOI—duration of injury (years); Level—neurological level of injury; Grade—SCI severity according to International Standards for the Neurological Classification of SCI.

SCI ID	Gender	Age	Trauma/NT	DOI (Years)	Level	Grade	Baclofen Use (Oral)
1	M	64	T	35	C4	D	No
3	M	54	T	13	C5	C	No
5	F	22	NT	1.5	C5	C	Yes
12	M	43	T	2	C4	D	Yes
15	M	56	T	20	C7	D	No
16	M	71	T	1.5	C3	D	Yes
17	M	54	T	3	C5	D	No
18	M	38	T	13	C3	C	No
19	F	62	T	4	C3	D	No
23	M	32	T	2	C5	C	No
25	M	26	T	3	C3	B	No
27	F	34	T	2	C3	A	Yes
28	M	63	T	4	C3	C	Yes
AB ID							
2	M	46					
6	M	22					
7	M	55					
8	M	58					
9	F	52					
10	M	47					
11	M	60					
13	F	22					
14	M	22					
20	M	24					
21	M	45					
22	M	26					
24	M	24					
26	M	51					
29	F	27					

**Table 3:** Responses to unconditioned cortical and spinal stimuli.

Participant	Group	TMS RMT	TMS120 Ampl	TSCS RMT
2	AB	32.0	0.848	27.0
6	AB	38.0	0.714	26.3
7	AB	35.0	0.696	24.3
8	AB	46.0	0.581	45.0
9	AB	35.5	0.289	26.2
10	AB	30.0	0.665	30.8
11	AB	43.5	0.383	45.0
13	AB	35.0	0.511	4.0
14	AB	49.5	0.413	22.3
20	AB	41.0	0.176	16.7
21	AB	40.5	0.466	10.2
22	AB	41.5	0.518	26.7
24	AB	46.0	0.801	21.7
26	AB	52.0	0.758	24.5
29	AB	44.5	0.415	25.7
**AB**	**Mean**	**40.7**	**0.549**	**25.1**
	SEM	1.7	0.051	2.8
1	SCI	53.0	0.182	15.0
3	SCI	53.5	0.093	33.3
5	SCI	30.5	0.261	5.8
12	SCI	62.0	0.058	27.8
15	SCI	34.0	0.713	38.0
16	SCI	72.0	0.065	38.7
17	SCI	51.0	0.182	42.5
18	SCI	36.5	0.074	23.0
19	SCI	61.0	0.186	4.8
23	SCI			40.1
25	SCI	71.0	0.058	33.7
27	SCI			18.7
28	SCI	50.0	0.142	23.2
**SCI**	**Mean**	**52.2**	**0.183**	**26.5**
	SEM	4.2	0.057	3.5

TMS: transcranial magnetic stimulation; RMT: Resting motor threshold; TSCS: Transcutaneous spinal cord stimulation. Mean values for each group listed in boldface.

**Table 4:** TMS responses to conditioning TSCS pulses.

Group	TSCS Intensity (% RMT)	Synapse Delay	Compared to TMS Alone (%)	SEM
AB	50%	−25	0.2%	9.4
		−10	15.4%	16.7
		−2	0.6%	8.9
		0	−11.4%	8.9
		2	−0.5%	7.1
		5	8.1%	16.2
		10	8.7%	9.1
		n/a	−98.9%	0.6
	70%	−25	−2.7%	4.8
		−10	2.9%	11.0
		−2	0.7%	6.3
		0	4.4%	7.8
		2	12.6%	11.2
		5	9.4%	12.2
		10	20.4%	19.6
		n/a	−97.4%	1.1
	90%	−25	−1.8%	13.3
		−10	4.7%	9.6
		−2	10.6%	10.5
		0	15.5%	13.9
		2	6.1%	8.9
		5	3.1%	9.8
		10	3.7%	6.6
		n/a	−93.1%	1.9
	n/a	n/a	0.0%	0.0
SCI	50%	−25	2.5%	6.4
		−10	4.7%	8.7
		−2	−1.1%	6.4
		0	−4.5%	4.4
		2	13.3%	12.9
		5	−9.1%	6.1
		10	0.6%	4.7
		n/a	−94.5%	2.1
	70%	−25	−10.1%	6.6
		−10	1.5%	7.3
		−2	−2.9%	6.5
		0	5.8%	5.4
		2	7.6%	12.8
		5	9.6%	8.1
		10	17.8%	8.2
		n/a	−92.0%	3.1
	90%	−25	−15.3%	4.5
		−10	12.1%	9.0
		−2	16.2%	21.8
		0	5.1%	7.6
		2	32.0%	27.8
		5	36.4%	24.3
		10	28.8%	21.9
		n/a	−88.2%	3.1
	n/a	n/a	0.0%	0.0

TSCS: Transcutaneous spinal cord stimulation; RMT: Resting motor threshold; n/a: not applicable.

**Table 5:** F-wave responses to conditioning TSCS pulses.

Group	TSCS Intensity (% RMT)	Synapse Delay	Ampl Compared to F Alone (%)	SEM	Persistence Compared to F Alone (%)	SEM
AB	50%	−200	23.1%	11.56	8.9%	6.0
		−50	6.7%	8.6	5.7%	6.3
		−25	1.7%	8.3	−2.8%	6.6
		−10	7.2%	7.2	−4.3%	5.5
		−5	−3.8%	11.6	−5.8%	5.5
		−2	12.0%	10.8	0.3%	5.0
		0	0.8%	7.3	1.8%	6.0
		n/a	−92.8%	2.1	n/a	n/a
	70%	−10	4.3%	7.7	−2.6%	8.0
		−5	16.8%	12.4	9.6%	10.1
		−2	5.8%	9.9	0.2%	5.9
		n/a	−77.4%	6.5	n/a	n/a
	175%	−10	−18.5%	14.6	−27.0%	9.3
		0	328.5%	136.1	39.5%	18.6
		n/a	1229.1%	340.4	n/a	n/a
	n/a	n/a	0.0%	0.00	0.0%	0.00
SCI	50%	−200	9.5%	7.7	6.0%	2.9
		−50	−1.5%	11.6	11.2%	8.9
		−25	−11.0%	9.0	−4.5%	9.4
		−10	−11.5%	8.9	−7.2%	8.0
		−5	−1.9%	6.4	5.7%	3.1
		−2	17.2%	16.9	9.2%	7.2
		0	−9.9%	11.3	−9.9%	9.8
		n/a	−77.7%	9.5	n/a	n/a
	70%	−10	−17.6%	6.5	−5.8%	4.0
		−5	−11.2%	4.5	−5.2%	2.9
		−2	−6.7%	6.2	3.1%	4.9
		n/a	−81.8%	7.7	n/a	n/a
	175%	−10	−27.7%	22.1	−7.0%	37.0
		0	371.4%	189.1	75.4%	35.8
		n/a	1649.4%	604.7	n/a	n/a
	n/a	n/a	0.0%	0.00	0.0%	0.00

TSCS: Transcutaneous spinal cord stimulation; RMT: Resting motor threshold; n/a: not applicable.

## Data Availability

The individual-level participant data presented in this study are available in the Supplementary Material and on Figshare at https://figshare.com/articles/dataset/dx_doi_org_10_6084_m9_figshare_6025748/6025748 (accessed on 4 October 2021).

## Supplementary Materials

The following are available online at https://www.mdpi.com/article/10.3390/jcm10225304/s1, Figure S1: Effects of TSCS on change in heart rate and blood pressure, Table S1: H-reflex responses to conditioning TSCS; Table S2: TSCS thresholds by individual and session; Table S3: TMS thresholds by individual and session; Table S4: F-wave amplitude and persistence by individual and session; Table S5: H-reflex amplitude by individual and session; Table S6: TSCS-TMS pairing by individual and session; Table S7: TSCS-F pairing by individual and session; Table S8: TSCS-H pairing by individual and session.
