# Supplementary figures and images for: Posteroanterior Cervical Transcutaneous Spinal Cord Stimulation: Interactions with Cortical and Peripheral Nerve Stimulation
Source: J Clin Med. 2021 Nov 15;10(22):5304. doi: 10.3390/jcm10225304 (PMC8623612; doi:10.3390/jcm10225304)

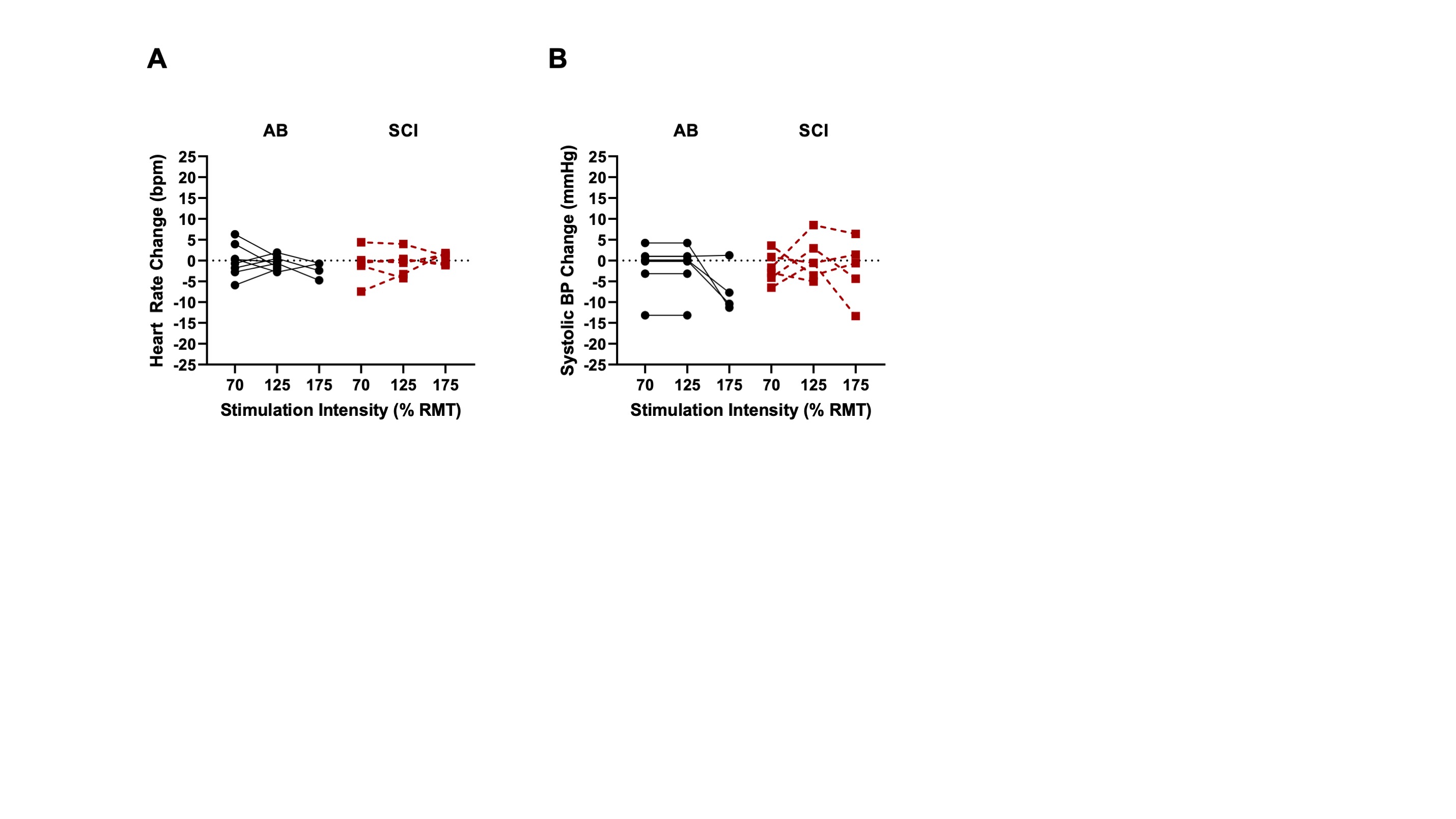

Supplement: Supplementary file 1 [file jcm-10-05304-s001.zip › Figs/Fig S1.jpg]
